# Development of a New Polymeric Nanocarrier Dedicated to Controlled Clozapine Delivery at the Dopamine D_2_-Serotonin 5-HT_1A_ Heteromers

**DOI:** 10.3390/polym13071000

**Published:** 2021-03-24

**Authors:** Sylwia Łukasiewicz

**Affiliations:** Department of Physical Biochemistry, Faculty of Biochemistry, Biophysics and Biotechnology, Jagiellonian University, 30-387 Krakow, Poland; sylwia.lukasiewicz@uj.edu.pl; Tel.: +48-012-664-61-34; Fax: +48-012-664-6902

**Keywords:** encapsulation, clozapine, schizophrenia, polymeric nanocarriers, D_2_-5-HT_1A_ receptor heterodimers, scFv antibodies

## Abstract

Clozapine, the second generation antipsychotic drug, is one of the prominent compounds used for treatment of schizophrenia. Unfortunately, use of this drug is still limited due to serious side effects connected to its unspecific and non-selective action. Nevertheless, clozapine still remains the first-choice drug for the situation of drug-resistance schizophrenia. Development of the new strategy of clozapine delivery into well-defined parts of the brain has been a great challenge for modern science. In the present paper we focus on the presentation of a new nanocarrier for clozapine and its use for targeted transport, enabling its interaction with the dopamine D_2_ and serotonin 5-HT_1A_ heteromers (D_2_-5-HT_1A_) in the brain tissue. Clozapine polymeric nanocapsules (CLO-NCs) were prepared using anionic surfactant AOT (sodium docusate) as an emulsifier, and bio-compatible polyelectrolytes such as: poly-l-glutamic acid (PGA) and poly-l-lysine (PLL). Outer layer of the carrier was grafted by polyethylene glycol (PEG). Several variants of nanocarriers containing the antipsychotic varying in physicochemical parameters were tested. This kind of approach may enable the availability and safety of the drug, improve the selectivity of its action, and finally increase effectiveness of schizophrenia therapy. Moreover, the purpose of the manuscript is to cover a wide scope of the issues, which should be considered while designing a novel means for drug delivery. It is important to determine the interactions of a new nanocarrier with many cell components on various cellular levels in order to be sure that the new nanocarrier will be safe and won’t cause undesired effects for a patient.

## 1. Introduction

Currently, the use of nanotechnology in molecular pharmacology has been attracting more and more attention. One of the leading trends in nanomedicine is the attempt to use drugs attached to nanoparticles in the therapy of many diseases. The nanoparticles delivery systems of active compounds create new possibilities, allowing, among other benefits, us to achieve a therapeutic effect only in a selected, well-defined target site, thus leading to the reduction or elimination of undesired side effects mainly related to non-selective action [1,2]. The main advantages of nanocarriers are their sub-cellular dimensions and tissue-cellular biocompatibility. The improvement of the compatibility of lipophilic, poorly water-soluble or even water-insoluble active compounds or increased drug permeability and absorption has been shown for various nanoformulations [3,4,5,6]. Drug encapsulation extends the duration of its action, protecting against rapid uptake and degradation, and by controlling the released dose it allows for maintaining concentration of the drug at the level of the required therapeutic concentration. Thus, such a strategy allows for reducing the size and frequency of doses [4,5,6,7]. In addition, the appropriate functionalization of the surface of the nanocarrier enables so-called “intelligent targeting”, i.e., release of the drug at the appropriate destination [5,6,8,9]. Thanks to this, nanotherapeutics have the ability to “perform complex operations” in the right place at the right time in the patient’s body, contrasting with previously developed preparations, whose greatest weakness has been non-selectivity. All of the above-mentioned features have consequently led to a reduction of the negative side effects of the therapy. Polymeric nanoparticles (PNp) are formed by biocompatible and biodegradable polymeric materials. This strategy enables a predictable decomposition process and complete metabolization of degradation products [10]. Polymeric nanocapsules (NCs) usually include an active compound immersed in a liquid core surrounded by a polymeric shell, which can be obtained from natural (chitosan, dextran, alginate, heparin, dextran sulfate, cellulose sulfate etc.) or synthetic polymeric (PLGA—poly(lactic-co-glycolic acid), PLA—poly(lactic acid), PGA—poly(glycolic acid), PCL—poly(caprolactone), PEI—poly(ethyleneimine), PLL—poly(l-lysine) etc.). To avoid serum protein adsorption, nonspecific binding to undesired cells and tissues, an outermost shell of the capsule is pegylated (grafted by polyethylene glycol–PEG). Selective delivery of active compounds requires the proper modification of a capsule shell, to which external elements (so-called targeting ligands) are embedded to obtain molecular recognition at the desired target location. One of the best targeting ligands are monoclonal scFv antibodies fragments (single chain variable fragments), which, due to their relatively small size, higher (as compared to other types of ligands) binding specificity as well as the lack of complement activating region and Fc domain (which directly translates into a reduction in the immunogenicity of the modified nanocapsules) [8,9,10,11], are increasingly used to functionalize nanocarriers. Moreover, the aptamer-functionalized nanocapsules show a good compatibility with the bloodstream and do not have a cytotoxic effect [5,6]. In conclusion, nanotechnology-based therapeutic methods provide unusual control over behaviour of the drug in the body, thus providing the possibility of targeted treatment.

Based on the current available literature, one may notice that in recent years there have been attempts at preparing a nanocarrier for antipsychotic drugs used to treat schizophrenia, a complex psychiatric disorder. Despite the better profile of atypical antipsychotics, they are not free of a negative impact on the patient’s organism. Both clinical and experimental studies indicate that the reduction of side effects is not complete, which is probably related to the non-specific action of the drug [12]. Therefore, the studies focusing on finding new agents with greater therapeutic efficacy are still in progress. The reference for this search is clozapine, which belongs to the group of atypical antipsychotic drugs. This drug is used in the clinic, however, and is not free from serious side effects such as: myocarditis, arrhythmia, weight gain, metabolic disorders, and above all agranulocytosis [13,14]. Due to risks of the above-mentioned complications, therapy with clozapine is often limited. The possibility to direct this compound to the desired site of action would greatly enhance its specificity.

Wang et al. (2020) produced clozapine containing solid lipid nanoparticles (SLNs) by using ultrasonic technology. They indicate that the encapsulation improves drug stability in the carrier system, and also increases drug bioavailability in vivo [15]. Ishak et al. (2013) showed different pharmacokinetic profile and biodistribution behavior of clozapine (CZP)-loaded NPs which were coated with chitosan, pluronic F-68, PEG 4000 and polysorbate 80 [16]. Moreover, the factors affecting drug encapsulation efficiency, particle size, surface charge, and surface hydrophilicity have been studied [17]. Additionally one can find some research that provides a brief summary and discussion of the progress and development in the delivery of other antipsychotics (e.g., aripiprazol, olanzapine, paliperidone) with nanoparticle formulations [18,19,20,21,22].

The main purpose of the manuscript is to cover a wide scope of the issues, which should be considered while designing this novel means for drug delivery. Very often scientific literature brings very enthusiastic information concerning the possibilities of modern nanomedicine, nano pharmacology and drug delivery, but the presented data are usually limited to the description of interactions of synthesized nanoparticles at the site of their destination, without data concerning interactions with cells of an immunological system, or possibilities of new nano carriers to turn on or promote the inflammation processes, genotoxicity, or, last but not least, data on in-vivo interactions. The main topic of the present review focuses on efforts which need to be undertaken to obtain a reliable, well-defined nanocarrier dedicated to drug delivery, in order to be sure that it will be safe and not cause undesired effects for a patient.

The present review covers a set of papers concerning studies leading to encapsulation of clozapine into polymeric nanocarriers. It is important to determine the interactions of new nanocarriers with many cell components on various cellular levels in order to be sure that the new nanocarrier will be safe and not cause undesired effects for a patient.

Considering the above issue, below is the description of the research which was undertaken to obtain an encapsulated form of clozapine. Clozapine polymeric nanocapsules (CLO-NCs) were prepared using anionic surfactant AOT (sodium docusate) as an emulsifier, and biocompatible polyelectrolytes such as: PGA and PLL. The outer layer of the carrier was grafted by PEG. Several variants of nanocarriers containing the antipsychotic varying in physicochemical parameters were tested. It seems that increasing the efficacy and safety of the clinical use of clozapine can be achieved by designing an appropriate nanocarrier to deliver the above-mentioned therapeutic to the selected target which constitutes the areas of the brain rich in dopamine D_2_ and serotonin 5-HT_1A_ (D_2_-5-HT_1A_) heteromers. The above strategy may contribute to increasing the availability and safety of the drug, as well as improve the selectivity of its action, resulting in increased effectiveness of schizophrenia therapy. However, the task is not easy and demands elaborate work, which is illustrated below.

### 1.1. Preparation of Polymeric Nanocapsules (NCs)

**Figure 1:**
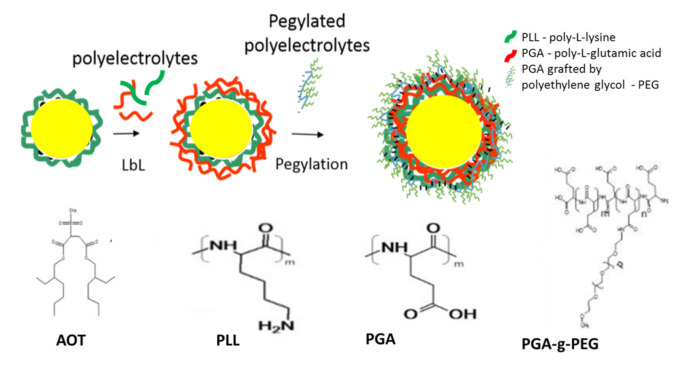
Structure of obtained polymeric NCs.

Polymeric nanocapsules (NCs) with a liquid core covered with a layer of biodegradable polyelectrolytes were prepared by the technique of sequential adsorption of the oppositely charged nanomaterials “layer-by-layer” LbL. The anionic surfactant AOT (sodium docusate, approved by FDA) as an emulsifier, and biocompatible polyelectrolytes such as: PGA polyanion, and PLL polycation were used. The pegylated outer layer of the capsule was prepared using PGA-g-PEG (PGA grafted by polyethylene glycol) (Figure 1). PEG is a neutral, hydrophilic polymer, and its high flexibility and mobility of the chain contributes to the stability of the NCs. Moreover, pegylated coatings are characterized by a reduced potential for protein adsorption, resulting in suppression of the opsonization process, and thereby a reduction of NCs uptake by cells of the immune system. The average size of the obtained NCs was 80–100 nm depending on the thickness of the outer layer. The obtained NCs were stable under physiological conditions at high ionic strength [23,24,25].

### 1.2. Interaction of the Nanocapsule/Target Cell—Cytotoxicity Studies of the Obtained Nanoformulations

**Figure 2:**
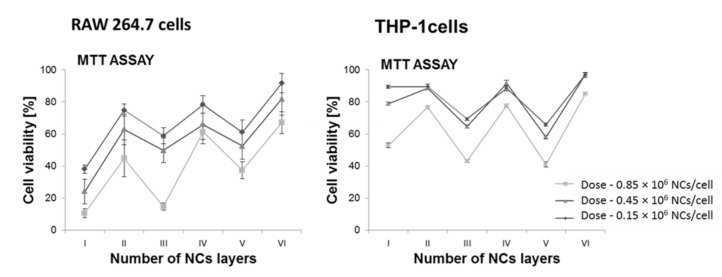
Cytotoxicity of obtained nanomaterials depending on their structure. Measurements for RAW 264.7 and THP-1 cells after a 24 h incubation with NCs. Detailed information [23,24].

Finding effective nanocarriers dedicated to the controlled delivery of active compounds requires systematic studies leading to the optimization of their interaction with target cells. This interaction depends on the type and physicochemical properties of the carrier and, above all, on the modification of its external layer. Quantification of cell viability allows to describe the toxicity of used nanomaterials. It is important to maintain a balance between the effective nanocarrier internalization and the induction of a toxic effect. The interaction between the NCs and the cell membrane is the main factor influencing this process and depends on the shape, size, flexibility, surface charge, modification and functionalization of a capsule [26,27,28,29]. In the case of NCs with a surface charge, their interaction with the cell membrane will be mainly determined by electrostatic interactions [26,27]. We have to take into account the fact that the sizes of capsules and their surface properties can significantly change in biological systems [30,31,32,33]. Due to varying ionic strength, as well as possible reactions with medium components (e.g., protein adsorption) spontaneous aggregation of NCs may occur [33,34,35,36]. Therefore, firstly, the biocompatibility and cytotoxicity of the obtained nanomaterials were determined, depending on the NCs dose, charge, size and modification of the outer layer. The experiments were carried out for various cell lines: HEK 293 (human embryonic kidney cell line), RAW 264.7 (mouse murine macrophages cell line), THP-1 (human leukemic monocyte cell line). The detailed results were presented in the publications [23,24]. Literature reports indicate a discrepancy in the obtained data using various tests dedicated to the estimation of cell viability [37,38]. Therefore, in order to obtain the most reliable results and avoid possible overinterpretation, several different tests were performed [23,24]. Generally, three main trends have been observed. Cytotoxicity depended on: (1) NCs concentration: for each type of tested NCs, the most toxic doses were defined, although it should be emphasized that for the most toxic types of NCs, the cell survival increased to 90% when the dose was reduced to 0.2 × 10^6^ NCs per cell, which is much above the assumed theoretical amount of NCs sufficient to achieve a therapeutic effect; (2) the number of polyelectrolyte layers—the smaller the number of layer (when we compere layers with the same charge—even or odd number of layers), the greater the decrease in cell viability; (3) surface charge—the negative charge on the NCs surface was correlated with increased survivability. Moreover, the obtained results indicate a relationship between the surface charge of the NCs and the destabilization of the cell membrane. In conclusion, the more toxic ones turned out to be positively charged NCs. Below (Figure 2) an example of the distribution of cytotoxicity depending on number of layers forming the nanocarrier, measured in RAW 264.7 and THP cells after 24 h incubation with NCs. More detailed information concerning the issue can be found in [23,24].

The mechanism of the observed phenomenon is probably similar for all tested cell lines and may be associated with a more efficient adsorption of positively charged NCs on the cell surface, a tendency to reduce lipid density and eventually disruption of cell membrane function. Modifications of the external layer involving the PEG grafting have a positive effect on cell viability (no toxic effects were observed). Moreover, pegylation spatially stabilized the NCs and prevented their aggregation [23,24].

### 1.3. Interactions of the Obtained NCs with Cells of the Immune System

The use of nanotechnology in the development of new controlled drug delivery systems also requires extensive studies on the interaction of nanomaterials with cells of the immune system. Numerous reports point to the rapid elimination of nanocarriers from the blood stream [39]. Adsorption of plasma proteins on the surface of the nanocarriers allows macrophages of the mononuclear phagocytic system (MPS) to quickly recognize and remove NCs before they reach their destination [40]. This translates directly into reducing the half-life of the drug and thus limits the ability of nanomaterials to function as efficient nanocarriers. Therefore, it is extremely important to design a nanocarrier that is non-visible for phagocytic cells and at the same time has all the features allowing for performing the required function. As was mentioned previously, the interaction of nanocarriers with target cells mainly depends on the type and physicochemical properties of the nanocarrier. Therefore, in accomplishing the assumed goals, NCs were tested depending on their size and charge, as well as modification of the outer layer. Numerous studies indicate that the appropriate modification of the outer layer has the greatest impact on interaction with phagocytic cells. Decorating the particle surface with a neutral hydrophilic polymer such as PEG blocks the electrostatic and hydrophobic interactions, which leads to the reduction or complete elimination of protein adsorption, thereby minimizing the opsonization process, which in consequence increase the lifetime of the nanocarrier in the bloodstream. This effect is correlated with the PEG properties. The proper pegylation of the particle surface is a crucial step, because the PEG quality, chain size, number of chains, density and the way they are arranged have a huge impact on the interaction with the target cell and biodistribution of the nanocarrier in the body [41]. In summary, the formation of a hydrophilic shell around the NCs protects it against rapid phagocytic uptake. On the other hand, pegylation may also intensify the internalization process of nanomaterials by other cell types (e.g., tumor cells or blood-brain barrier cells) [39,42]. Reports indicate that both phagocytosis, endocytosis and micropetrocytosis may be involved in the internalization process [1]. Considering the above issues, the conducted experiments also focused on the study of the interaction between the obtained NCs (with different physicochemical parameters) and cells of the immune system. RAW 264.7 and THP-1 cell lines, as well as human monocyte-derived macrophages (HMDMs) cells that were differentiated from peripheral blood mononuclear (PMBC) cells from healthy donors were used in our studies [24,43]. It has been shown that all types of synthesized NCs are taken up by phagocytic cells; however, the uptake of pegylated NCs was substantially lower compared to unmodified NCs. The strongest inhibition of the process was observed in the case of blocking (in experiments specific inhibitors for specific endocytosis pathways were used) clathrin mediated endocytosis (RAW 264.7, THP-1). The presence of all types of obtained NCs in lysosomes was also visualized. In addition, it has been shown that unmodified NCs, in contrast to pegylated NCs, have an influence on the phagocytic potential. None of the obtained NCs variants also led to the differentiation of THP-1 cells. Based on the above observations, it was concluded that the obtained polymeric NCs can be successfully modified (by PEG grafting) in a way that allows them to be masked for phagocytic cells. This confirmed the earlier hypothesis that synthesized NCs are a promising candidate that can be used for controlled drug delivery (more detailed information concerning the issue one can find in [24,43]).

### 1.4. Obtaining and Characterization of the Encapsulated Form of Clozapine

**Figure 3:**
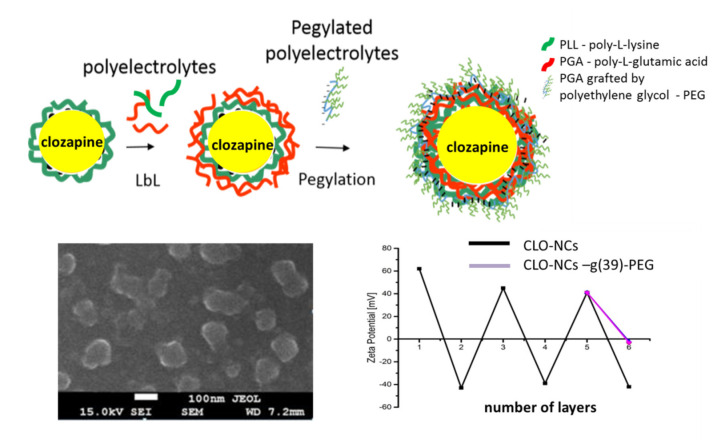
**Upper panel**—structure of CLO-NCs obtained using LbL technique. **Lower panel**—SEM micrograph of CLO-NCs VI PGA-g(39)-PEG and zeta potential measurements. Detailed information [25].

Currently, encapsulation of active compounds is a promising strategy in modern molecular pharmacology. Therefore, in the light of the above-described issues, encapsulation of clozapine, allowing its controlled release and delivery, can lead to an improvement of the therapeutic potential of the drug, which may have a direct impact on the quality of schizophrenia therapy. Based on the data obtained in previous experiments, the type of nanocarrier used to encapsulate clozapine was defined [23,24]. These were six-layer polymer pegylated NCs. The polyelectrolyte layer (PLL/PGA) was formed by the sequential adsorption (LbL) method on the emulsion core, which contained dissolved clozapine. Several variants of capsules containing the above-mentioned drug, differing in physicochemical parameters (thickness of the outer layer, pegylation, charge) were obtained. Respectively, they were: positively charged five-layered NCs—CLO-NCs V-PLL, negatively charged six-layered NCs—CLO-NCs VI-PGA and neutral, pegylated six-layer NCs—CLO-NCs VI-PGA-g(x)-PEG (different PEG grafted). The synthesis, physicochemical properties stability, as well as he release profile of clozapine of the obtained NCs are well described in [25]. Schematic representation of prepared CLO-NCs is illustrated in the Figure 3.

**Figure 4:**
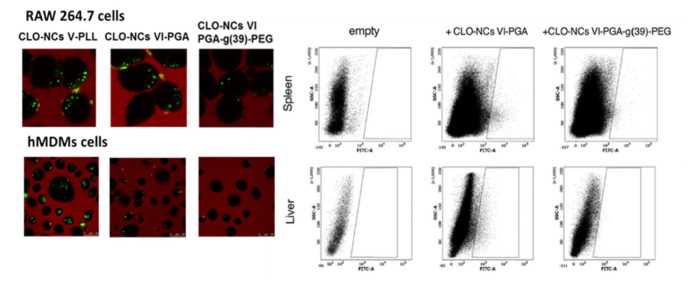
**Left panel**—In vitro CLO-NCs internalization studies performed in RAW 264.7 and hMDMs (human monocyte-derived macrophages) cells after a 2 h incubation with NCs. **Right panel**—In vivo-CLO-NCs biodistribution studies: the animals were injected with 150 mL suspension of CLO-NCs VI-PGA as well as CLO-NCs VI-PGA-g(39)-PEG. Flow cytometry studies were performed 4 h after injection. Detailed information [25].

Based on the experience collected during the study of empty carriers, similar experiments were carried out to determine the behavior of carriers with encapsulated clozapine. Cytotoxicity and cell viability studies as well as interaction with phagocytic cells (Figure 4) showed similar results compared to those obtained with empty carriers [25]. CLO-NCs VI-PGA-g (39)-PEG was the formulation with the best parameters.

While designing new drug carriers it is extremely important to estimate their biodistribution in an in-vivo system. Although the experiments with the use of mice were only qualitative, they clearly indicated dependence of the biodistribution profile on the modification of the outer layer of the capsule. Four hours after injection, the presence of CLO-NCs VI-PGA was confirmed mainly in the mouse liver and spleen (Figure 4), and on a smaller scale in the kidneys and lungs. Pegylation of the outer layer (CLO-NCs VI-PGA-g(39)-PEG) significantly reduced the accumulation level of capsules in the investigated organs [25].

Moreover, behavioral studies of the effectiveness of the encapsulated form of clozapine in experimental animals were performed [25]. The obtained results show that the encapsulated clozapine reduced the locomotor activity of mice in a manner characteristic of free clozapine; however, this effect was induced only by pegylated CLO-NCs (CLO-NCs VI-PGA-g(39)-PEG). Unpegylated NCs were not effective, probably due to their rapid elimination by macrophages. Although the obtained results are qualitative the effects of clozapine at significantly lower doses have been observed at this stage. In conclusion, the obtained results indicate the validity of clozapine encapsulation [25].

### 1.5. NCs Interactions with hCMEC/D3 Cells (Immortalized Human Cerebral Endocrine Cells, D3 Clone) Constituting the In Vitro Model of the Human Blood-Brain Barrier

Designing new drug delivery systems, especially those directed to the brain areas, it is first of all necessary to find an answer whether the new nanocarrier will be able to cross the blood brain barrier (BBB). Currently, several cell lines which have the characteristic of cells forming this natural barrier have been derived [44,45]. The best known in-vitro model of human BBB is the hCMEC/D3 cell line. This line was derived through the immortalization of human primary brain endothelial cells [45]. hCMEC/D3 cells show a morphology similar to primary cells, they form tight junction, exhibit trans-endothelial electrical resistance (TEER) and also maintain important and characteristic features of BBB, such as: expression of junctional proteins and efflux transporters [44,45]. This cell line was a convenient model dedicated to the study of the transcytosis process [46].

As mentioned earlier, the quantification of cell viability is a key element in understanding the interaction between NCs and the target cell. Therefore, when starting experiments using the human BBB model, these kinds of experiments were performed [43]. Also in this case, several different tests were carried out. Additionally, an attempt to answer the question whether cell death occurs through necrosis or apoptosis was made. As in the case of other tested cell lines, a decrease in viability, depending on NCs concentration, was shown, although in the case of hCMEC/D3 cells the scale of this phenomenon was much smaller, which probably could be related to a well-developed efflux transport and thus the rapid removal of excess capsules from the cell. Numerous reports [27,29,34], including our earlier studies [23,24,25], indicate a correlation between the positive charge on the NCs surface and the decrease in cell viability. This effect, associated with disruption of the cell membrane, is probably a common feature of all positively charged nanomaterials. The high level of LDH release due to the stimulation of the five-layered NCs supports this hypothesis. Unfortunately, based on the obtained data, it cannot be unambiguously determined whether apoptosis or necrosis has its contribution to cell death. In summary, the most promising results were obtained for six-layer capsules with a pegylated outer layer (CLO-NCs VI-PGA-g(39)-PEG), where the cell viability was almost 100%, after 24 and 48 h incubation with NCs.

BBB is an anatomical-functional system that regulates the exchange of substances between blood and the central nervous system (CNS). BBB maintains optimal homeostasis and protects of CNS against harmful substances, as well as enables selective transport of compounds circulating in the blood into the cerebrospinal fluid [47,48,49]. Due to the precise selectivity of the barrier, transport of therapeutic compounds to the brain is quite a challenge, because only uncharged, lipophilic and relatively small sizes substances can pass through the BBB without major obstacles. These are serious limitations that cannot be managed by currently available therapeutics [50]. Additional restrictions are precise transport mechanisms in endothelial cells, i.e., low level of pinocytic vesicles and selective transporters in the cell membrane [50]. Moreover, endothelial cells that have a polarized membrane to transport use transcytosis process (endocytosis on the apical side and exocytosis on the basolateral side) [47]. Therefore, the development of nanocarriers used to deliver drugs to defined areas of the brain requires detailed study of the transport process across BBB. Various endocytic pathways used for the internalization of exogenous substances by endothelial cells have been described in the literature [47,51]. Therefore, several experiments were carried out in order to find answers to three basic questions: (1) whether the mechanism of internalization is energy-dependent, (2) whether it occurs via endocytosis and (3) if yes, which endocytosis pathway is involved in the process. The obtained results [43] indicate the dependence of the process on NCs dose and time. The highest level of internalization has been described for positively charged, non-pegylated NCs, which is probably related to the facilitated interaction between the capsule and the cell membrane [34]. Moreover, the obtained results suggest an energy- and clathrine-dependent mechanism of internalization for all tested types of NCs and additional passive transport in the case of pegylated NCs. Confocal microscopy studies indicate the presence of synthesized NCs within clathrine vesicles as well as in the early endosomes and lysosomes. Considering the use of a new carrier for clozapine, it was necessary to carry out experiments showing not only the ability of model cells to internalize NCs, but also the ability of obtained NCs to cross the BBB.

**Figure 5:**
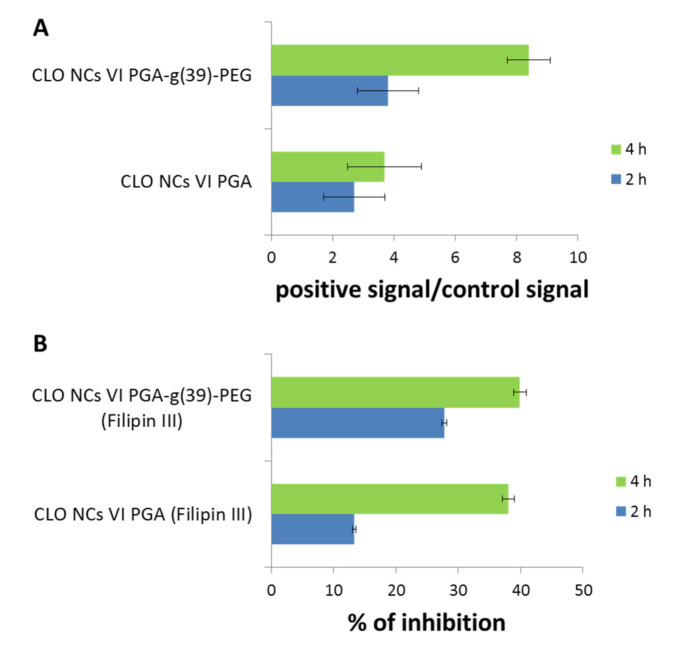
(**A**) Transcytosis experiment performed in hCMEC/D3 cells for CLO-NCs VI PGA and CLO-NCs VI PGA-g(39)-PEG (transwell pore-3mm). (**B**) Inhibition of the transcytosis process (incubation with filipin III—specific inhibitor of the process) for various types of CLO-NCs (detailed information [43]).

In-vitro experiments involving hCMEC/D3 cell line revealed that the observed transcytosis process depended on NCs dose and time and the strongest effect was recorded for pegylated CLO-NCs (CLO-NCs VI-PGA-g(39)-PEG)—detailed information [43]. The most important data are presented in the Figure 5 where one can observe significant increase of transcytosis process (after 4 h incubation time) in case of pegylated CLO-NCs in comparison to PEG-unmodified CLO-NCs. Studies using a specific transcytosis inhibitor (filipin III) point to caveolae-dependent mechanism of the process. In conclusion, CLO-NCs VI-PGA-g(39)-PEG are able to cross the BBB and represent a promising model of the nanocarrier for clozapine.

### 1.6. Heteromer of the D2-5-HT1A Receptors as an Important Target for Clozapine

The concept of oligomerization of G-protein coupled receptors (GPCRs) plays an important role in modern molecular pharmacology. The physical association of receptor proteins indicates a new level of signal complexity and the possibility of changing the pharmacological properties of the receptors included in the complex [52,53,54]. Research aimed at finding therapeutic substances operating via selective recognition of GPCRs heteromers is being undertaken more and more often. Such a strategy allows us to obtain a tissue-specific effect, since the interaction between receptors engaged in the complex formation can only take place when the receptors are simultaneously expressed on the same cell. Many recent reports indicate the existence of clinically relevant GPCRs heteromers, important in the treatment of, among others, pain, asthma or Parkinson’s disease [54,55,56,57,58]. This evidence shows that GPCRs heteromers constitute extremely important targets in the design of modern treatment routes.

Both D_2_R and 5-HT_1A_R are important sites of action of atypical antipsychotics [59], hence studies on the interaction between these receptors in the context of antipsychotic activity have been undertaken.

**Figure 6:**
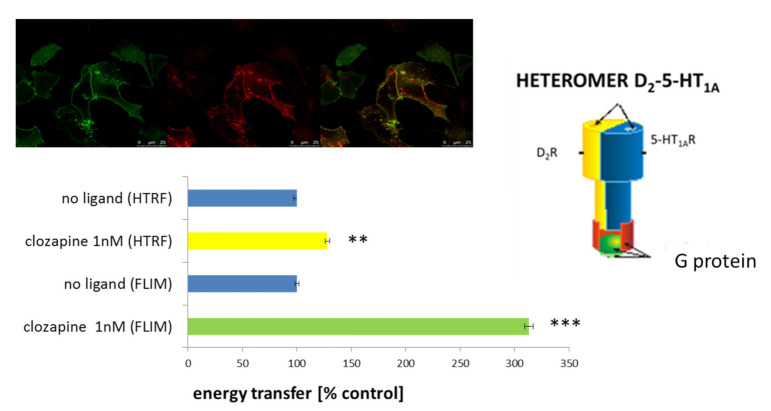
Constitutive dimerization of dopamine D_2_ and serotonin 5-HT_1A_ receptors. **Upper panel**—HEK 293 cells expressing the dopamine D_2_ (red) and serotonin 5-HT_1A_ (green) receptors; colocalization of both receptors (yellow). **Lower panel**—bar graph presenting FRET (fluorescence resonance energy transfer) measurements using HTRF (homogenous time resolved FRET) and FLIM (fluorescence life time imagine microscopy) techniques. The statistical significance was evaluated using student t-test and Mann–Whitney U-test, ** *p* < 0.01, *** *p* < 0.001. **Right panel**—schematic presentation of D_2_-5-HT_1A_ heteromer. Detailed information [60].

The obtained results are discussed in more detail in [60]. Constitutive dimerization of both investigated receptors in HEK 293 cells has been shown for the first time. To avoid the possibility of data misinterpretation, D_2_-5-HT_1A_ receptor heteromers were confirmed by two independent techniques based on monitoring FRET (fluorescence resonance energy transfer) phenomenon (FLIM—fluorescence life time imagine microscopy and HTRF—homogenous time resolved FRET) (Figure 6). Moreover, the effect of various antipsychotics (inter alia: clozapine, aripiprazole, 8-OH-DPAT) on the heteromerization process has been determined. The highest effect was observed after incubation of cells with clozapine (Figure 6), aripiprazole and simultaneous administration of clozapine and 8-OH-DPAT [60]. In the previous studies, [61,62,63,64], the opposite to the above-described effects of clozapine for dopamine D_1_ (D_1_R) and D_2_R (D_1_-D_2_) heteromers, as well as serotonin 5-HT_2A_ (5-HT_2A_R) and D_2_R (D_2_-5-HT_2A_) heteromers, have been shown. These data indicated the specific effect of clozapine, depending on the type of receptors forming the complex. Furthermore, in vivo D_2_R and 5HT_1A_R co-localization in the mouse prefrontal cortex has been shown [60]. It suggests a potential presence of the above-mentioned heteromers in the brain. Additionally, to estimate the activation of intracellular signal transduction pathways as a result of antipsychotic action on D_2_-5-HT_1A_ heteromers several functional tests have been performed. The experiments were carried out in HEK 293 cells expressing these receptors in various combinations. This approach enabled differentiation of the action of investigated compounds, depending on the presence of homo- or heteromeric complexes. Although the functional consequences of signal transmission via D_2_-5-HT_1A_ heteromers are still not fully explained, the studies indicate the initiation of different signalling pathways depending on whether the receptors are co-expressed or produced individually in a cell. In summary, these results point to the possibility of antipsychotic action by specific targeting of active compounds on D_2_-5-HT_1A_ heteromers (which have been shown to be present in cortical neurons [60] and they can also be an inspiration to improved pharmacotherapy of schizophrenia.

### 1.7. Synthesis of a Targeting Ligand Specifically Recognizing the D2-5-HT1A Heteromer for Functionalization of the Obtained CLO-NCs VI-PGA-g(39)-PEG

The next step was the decorating of NC surfaces by attaching targeting ligands, which would allow selective delivery of drugs to defined target sites. Among others, human monoclonal antibody fragments—scFv (single-chain variable fragment) can be successfully used as targeting ligands. These antibodies consist of variable heavy (VH) and light (VL) regions of immunoglobulin chains linked by an elastic peptide linker designed to allow contact between the two chains and preserve the antigen binding site within a single linear molecule [57]. ScFvs fragments, compared to larger forms of monoclonal antibodies such as: Fab, F(ab)_2_, IgG, are characterized by lower retention time in non-target tissues, better tissue penetration and reduced immunogenicity, which makes them attractive candidates for therapeutic applications [58].

**Figure 7:**
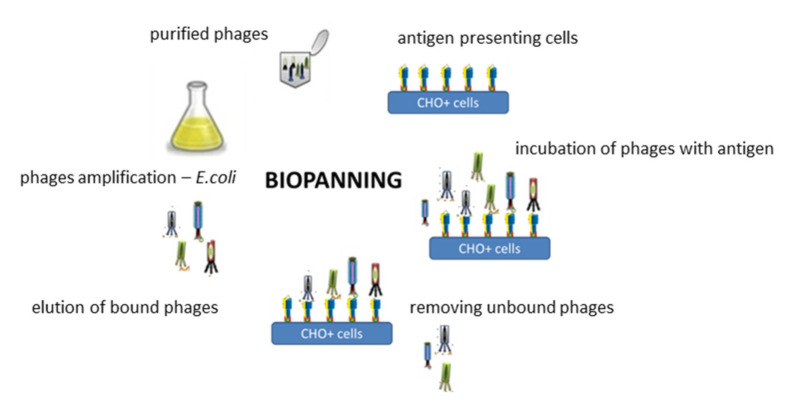
Phage display technique—biopanning process. For the isolation of phages specifically recognizing the D_2_-5-HT_1_A heteromer, the immune-selection rounds were performed. Purified phages were incubated with CHO+ line cells (CHO-K1 stable line) overexpressing both types of desired receptors. In the next step unbounded phages were removed in the process of intensive rinsing. Then the selected phages which point to affinity to D_2_-5-HT_1A_ heteromer were eluted, amplified, purified and used in the next round of the positive selection. Detailed information [66].

Based on the data described above [60] we aimed to develop a targeting ligand in the form of a fragment of a human monoclonal scFv antibody specifically recognizing the D_2_-5-HT_1A_ heteromer. To fulfil its role, such antibody must recognize the structural epitope formed within the heteromeric structure and, at the same time, not show specificity for monomeric or homomeric forms of the receptors. To accomplish this task the phage display technique—described for the first time by Smith [65]—Nobel laureate in 2018—was adapted [66]. The phagemid library of human scFv antibodies Tomlinson I + J (Geneservice) was used. This library allows for the preparation of approximately 3 × 10^8^ different phages, the envelope of which the PIII-scFv fusion protein encoded by the pIT2 phagemid is embedded. Since both receptors included in the heteromer belong to the family of membrane proteins, it was extremely important to carry out the selection rounds, so-called bio-panning (Figure 7), under conditions most similar to those in which these receptors occur naturally in the cells, which allowed to preserve the native spatial conformation of the heteromer. For the isolation of phages specifically binding to the D_2_-5-HT_1A_ heteromer, the immune-selection rounds were performed on CHO+ line cells (CHO-K1 stable line) overexpressing both receptors. Purified phages were incubated with antigen which constituted D_2_-5-HT_1A_ heteromer presented on CHO+ cells. Then, by intensive rinsing, the unbound phages were removed. In the next stage the selected phages which possessed affinity to D_2_-5-HT_1A_ heteromer were eluted, amplified, purified and used in the next round of the positive selection. Phages binding receptor monomers as well as other proteins present on the surface of CHO-K1 cells were eliminated by negative selection, using a CHO- cells. The CHO- cells constituted the mixture of stable CHO-K1 cells lines overexpressing only the single type of receptors forming the heteromer.

To obtain a soluble form of scFv antibodies in the *E. coli* HB2151 expression system, the monoclonal phages, isolated in the selection process, that most strongly bind to the defined heteromer were used. The purification procedures based on affinity chromatography using Protein L-immobilized resin was performed. As a result of the experiments, the scFv monoclonal antibody specifically recognizing the D_2_-5-HT_1A_ heteromers was isolated, and it has been used as a targeting ligand for functionalization of model NCs. The procedure is described in detail in [66].

## 2. Conclusions

**Figure 8:**
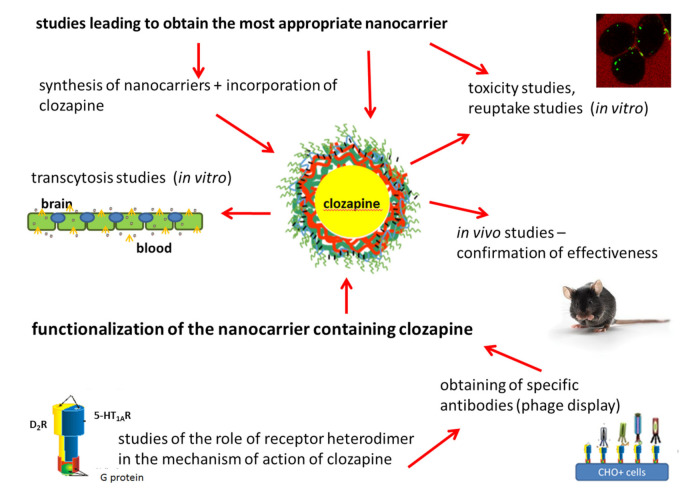
The full experimental paradigm.

The obtained NCs containing clozapine (CLO-NCs VI-PGA-g-PEG) represent a promising novel formulation of this compound. The encapsulated form of clozapine is safer since it does not influence the viability of diverse cells, does not cause activation of immunological system, and can cross BBB easily, not involving unsealing of the barrier. The experiments described here were carried out mainly using in-vitro models; however, preliminary studies showed that the CLO-NCs VI-PGA-g-PEG formulation allows clozapine effect in-vivo. In order to fully describe the behavior of nanocarriers, further detailed and extensive in-vivo studies are necessary. Moreover, the research points to the importance of modification of the most outer layer (surface) of the nanocarrier. NCs charge, pegylation process, as well as functionalization determining physico-chemical parameters of the nanocarriers enable its proper functioning. Table 1 includes summary of the results ob-tained for various variants of polymeric nanocapsules (NCs) constituting a new nanocar-rier for clozapine. As can be seen from the above review, each stage of the research towards obtaining the optimal nanocarrier is laborious and requires a lot of work. The full experimental paradigm is illustrated below (Figure 8). This kind of study engages specialists with wide knowledge in the field of chemistry, biochemistry, biophysics, biotechnology, molecular biology, pharmacology, molecular medicine etc. Designing of a nanocarrier from the chemical point of view, evaluation of its physico-chemical properties, description of its biochemical interaction in in vitro as well as in-vivo studies, designation of its pharmacological profile, and, last but not least, indication the desired site of action together with formulation of targeting ligand lead to professional development of new strategies of targeted delivery platform.

Each stage of the presented research leading to full characterization of the functionalized nanocarrier requires elaborative work and is very demanding. However, the effort is worth it, because clozapine (or other antipsychotic drug) encapsulating and directing its activities in the defined areas enables enhancing its selectivity and specificity, as well as limits side effects which undoubtedly may contribute to increasing the safety of the schizophrenia therapy.

## Figures and Tables

**Table 1:** Summary of the results obtained for various variants of polymeric nanocapsules (NCs) constituting a new nanocarrier for clozapine.

	NCs I/III-PLL ^b^	NCs II/IV-PGA ^c^	NCs V-PLL ^d^	NCs VI-PGA ^e^	NCs VI-PGA-g(39)-PEG ^f^	CLO-NCs V-PLL ^g^	CLO-NCs VI-PGA ^h^	CLO-NCs VI-PGA-g(39)-PEG ^i^	Ref.
Charge of outer layer of nanocarrier	Positive	Negative	Positive	Negative	Neutral	Positive	Negative	Neutral	[23,24,25]
Toxicity ^a^	High	Midium	Medium	Low	Low	Medium	Low	Very low	[23,24,25,43]
RAW and THP-1 cellls uptake	-	-	High +++ Clatrine path	High ++ Clatrine path	Low Clatrine path and passive transport	High +++ Clatrine path	High ++ Clatrine path	Low Clatrine path and passive transport	[24,25]
hMDMs cells Uptake	-	-	High +++	High ++	Low	High +++	High ++	Low	[43]
Phagocytic cells	Visible +++	Visible ++	Visible +++	Visible ++	Invisible	Visible +++	Visible ++	Invisible	[23,24,25,43]
hCMEC/D3 cells (in vitro BBB model) Uptake	-	-	High +++ Clatrine path	High ++ Clatrine path	High ++ Clatrine path and passive transport	High +++ Clatrine path	High ++ Clatrine path	High ++ Clatrine path and passive transport	[43]
Stimulation of phagocytic potential	-	-	-	-	-	High +++	High ++	Medium	[23,24,43]
Biodistribution In vivo	-	-	-	-	-	-	Lungs ++ Liver +++ Spleen +++ Kidney ++	Spleen + kidney +	[25]
Behavioral studies In vivo	-	-	-	-	-	-	Mice locomotor activity reductio, similarity to clozapine +	Mice locomotor activity reductio, similarity to clozapine ++	[25]
Transcytosis (hCMEC/D3 cells) In vitro BBB model)	-	-	High ++	High +	High +++	High ++	High + Caveole path	High +++ Caveole path	[43]

^a^ Similar results have been recorded for various cells lines using different assays (see references). ^b^ **NCs I/III-PLL**—one or three layers polymeric nanocapsules (NCs) prepared using anionic surfactant AOT (sodium docusate) as an emulsifier, and biocompatible polyelectrolytes such as: PGA (poly(glycolic acid)) and PLL (poly(l-lysine)). Outer layer of the carrier constitutes PLL. ^c^ **NCs II/IV-PGA**—two or four layers polymeric nanocapsules (NCs) prepared using anionic surfactant AOT (sodium docusate) as an emulsifier, and biocompatible polyelectrolytes such as: PGA (poly(glycolic acid)) and PLL (poly(l-lysine)). Outer layer of the carrier constitutes PGA. ^d^ **NCs V-PLL**—five layers polymeric nanocapsules (NCs) prepared using anionic surfactant AOT (sodium docusate) as an emulsifier, and biocompatible polyelectrolytes such as: PGA (poly(glycolic acid)) and PLL (poly(l-lysine)). Outer layer of the carrier constitutes PLL. ^e^ **NCs VI-PGA**—six layers polymeric nanocapsules (NCs) prepared using anionic surfactant AOT (sodium docusate) as an emulsifier, and biocompatible polyelectrolytes such as: PGA (poly(glycolic acid)) and PLL (poly(l-lysine)). Outer layer of the carrier constitutes PGA. ^f^ **NCs VI-PGA-g(39)-PEG**—six layers polymeric nanocapsules (NCs) prepared using anionic surfactant AOT (sodium docusate) as an emulsifier, and biocompatible polyelectrolytes such as: PGA (poly(glycolic acid)) and PLL (poly(l-lysine)). Outer layer of the carrier constitutes PGA grafted by PEG (polyethylene glycol), grafting percentage was 39%. ^g^ **CLO-NCs V-PLL**—five layers polymeric nanocapsules (NCs) containing clozapine, prepared using anionic surfactant AOT (sodium docusate) as an emulsifier, and biocompatible polyelectrolytes such as: PGA (poly(glycolic acid)) and PLL (poly(l-lysine)). Outer layer of the carrier constitutes PLL. ^h^ **CLO-NCs VI-PGA**—six layers polymeric nanocapsules (NCs) containing clozapine, prepared using anionic surfactant AOT (sodium docusate) as an emulsifier, and biocompatible polyelectrolytes such as: PGA (poly(glycolic acid)) and PLL (poly(l-lysine)). Outer layer of the carrier constitutes PGA. ^i^ **CLO-NCs VI-PGA-g(39)-PEG**—six layers polymeric nanocapsules (NCs) containing clozapine, prepared using anionic surfactant AOT (sodium docusate) as an emulsifier, and biocompatible polyelectrolytes such as: PGA (poly(glycolic acid)) and PLL (poly(l-lysine)). Outer layer of the carrier constitutes PGA grafted by PEG (polyethylene glycol), grafting percentage was 39%. ‘+’—reflects intensity of the marked process.
